# The Use of CRISPR-Cas Systems for Viral Detection: A Bibliometric Analysis and Systematic Review

**DOI:** 10.3390/bios15060379

**Published:** 2025-06-12

**Authors:** Othmane Jeddoub, Nadia Touil, Omar Nyabi, Elmostafa El Fahime, Khalid Ennibi, Jean-Luc Gala, Abdelaziz Benjouad, Lamiae Belayachi

**Affiliations:** 1 International Faculty of Medicine, Health Sciences Research Center, College of Health Sciences, International University of Rabat, Technopolis Parc, Rocade of Rabat-Salé, Sala-Al Jadida 11100, Morocco; 2 Unité de Recherche Biomédicale et Epidemiologie (URBE), Centre de Virologie, Maladies Infectieuses et Tropicales, Hopital Militaire d’Instruction Mohammed V, Faculté de Médecine et de Pharmacie de Rabat, Rabat 10100, Morocco; 3 Mohammed VI Center for Research and Innovation (CM6RI), Rabat 10100, Morocco; 4 Center for Applied Molecular Technologies (CTMA), Institute of Clinical and Experimental Research, Université Catholique de Louvain, 1200 Brussels, Belgium; omar.nyabi@uclouvain.be (O.N.); jean-luc.gala@uclouvain.be (J.-L.G.); 5 Mohammed VI University of Sciences and Health (UM6SS), Casablanca 20370, Morocco; 6 Immunopathology Research Team (ERIP), Faculty of Medicine and Pharmacy, University Mohammed V, Rabat 10100, Morocco

**Keywords:** bibliometric analysis, CRISPR-Cas systems, viral diagnostic assay, isothermal amplification

## Abstract

Viral infections impose a significant burden on global public health and the economy. This study examines the current state of CRISPR-Cas system research, focusing on their applications in viral detection and their evolution over recent years. A bibliometric analysis and systematic review were conducted using articles published between 2019 and 2024, retrieved from Web of Science, Scopus, and PubMed databases. Out of 2713 identified articles, 194 were included in the analysis. The findings reveal substantial growth in scientific output related to CRISPR-Cas systems, with the United States leading in research and development in this field. The rapid increase in CRISPR-Cas research during this period underscores its immense potential to transform viral diagnostics. With advantages such as speed, precision, and suitability for deployment in resource-limited settings, CRISPR-Cas systems outperform many traditional diagnostic methods. The concerted efforts of scientists worldwide further highlight the promising future of this technology. CRISPR-Cas systems are emerging as a powerful alternative, offering the possibility of expedited and accessible point-of-care testing and paving the way for more equitable and effective diagnostics on a global scale.

## 1. Introduction

The COVID-19 pandemic has underscored the critical importance of rapid and accurate diagnostics while exposing a widespread shortage in diagnostic capacities and resources [1,2,3]. Recent data indicate that 47% of the global population has limited or no access to essential diagnostics, including viral detection through antigen, antibody, or nucleic acid methods. This situation is particularly alarming in poor, rural, and marginalized communities, where healthcare infrastructure is insufficient for providing basic diagnostic services [4]. These deficiencies reaffirm the conclusions of *The Lancet’s* 2018 series on pathology and laboratory medicine in low- and middle-income countries [5], which highlighted the inequality of access to diagnostic testing, despite its central role in healthcare. The real challenge lies in facilitating mass testing and screening to identify infected individuals, particularly those with asymptomatic infections, to prevent the unintentional spread of viral infections [6,7]. Recent technological innovations offer significant potential to transform diagnostics, with a focus on point-of-care (POC) applications that deliver fast, high-quality testing in resource-limited communities, following the ASSURED criteria (Affordable, Sensitive, Specific, User-friendly, Rapid, Equipment-free, Delivered) established by the World Health Organization (WHO). It is crucial to develop diagnostics that balance affordability and accuracy, as low-cost tests, such as rapid antigen tests, often compromise sensitivity and specificity compared to more advanced laboratory tests [8,9].

Nucleic acid tests (NATs) remain the gold standard for diagnosing a wide range of chronic and acute diseases, particularly those caused by infectious agents such as viruses [10]. Polymerase chain reaction (PCR) is one of the most commonly used NAT methods, recognized for its accuracy. However, PCR requires sophisticated infrastructure and trained personnel, limiting its use in point-of-care settings without adequate laboratory facilities [10]. To address these limitations, a new technique called isothermal amplification has been developed. This method allows for the multiplication of nucleic acid sequences at a constant temperature, eliminating the need for thermal cycling [11]. It simplifies processes and reduces the need for sophisticated equipment, making diagnostics more accessible in point-of-care settings [9]. Among isothermal amplification techniques, two stand out due to their widespread use and current popularity: LAMP (loop-mediated isothermal amplification) and RPA (recombinase polymerase amplification). LAMP is a rapid and efficient method for amplifying DNA at a constant temperature, with sensitivity comparable to PCR. It utilizes the Bst DNA polymerase from Bacillus stearothermophilus, which exhibits high strand displacement activity at 60–65 °C, creating a dumbbell-like DNA structure and requiring four primary primers [11], with the option to add loop primers (LF/LB) [12] to enhance efficiency. By recognizing six distinct sequences initially and four additional sequences in later stages, LAMP provides high specificity while minimizing background interference. One of LAMP’s key advantages is its ability to efficiently amplify DNA at extremely low levels, often comparable to PCR, with detection limits down to just a few copies (up to 10^9^) of the target DNA [13]. Moreover, LAMP can be adapted for RNA detection (RT-LAMP) by incorporating reverse transcriptase, which transcribes RNA into cDNA, enabling the detection of RNA viruses such as SARS-CoV-2 (30), Japanese encephalitis virus 36, measles [14], Ebola [15], and monkeypox viruses [16]. Although LAMP is a highly robust technique, it does have certain limitations. The use of multiple primers in LAMP assays can increase the risk of non-specific amplification, which may result in false positives [17]. Additionally, LAMP is not ideal for amplifying short gene sequences [18]; the design of multiple primers for LAMP presents significant challenges for researchers, particularly when dealing with highly polymorphic sequences, due to the limited primer selection options available [19]. Another notable drawback is the difficulty of simultaneously detecting multiple targets in a single tube; this is largely due to the reliance on fluorogenic probes that contain target-specific sequences, making it more complex to adapt and optimize the assays for multiplex detection [20]. One of the challenges with LAMP is the inability to clone and sequence target products, as the amplified DNA forms complex loop structures that are not easily compatible with conventional cloning and sequencing methods [14]. Recent innovations have significantly improved the LAMP technique, including modifications to its components and the optimization of detection methods. These advancements are paving the way for more robust and efficient versions of LAMP for viral infection detection. Crego-Vicente et al. [20] explored various LAMP multiplexing methodologies, each with different operating conditions and mechanisms. While every methodology has its strengths and weaknesses, the choice depends largely on the specific application. Additionally, B. Jia et al. [21] developed GLAPD, a novel system for designing LAMP primers for a set of target genomes using whole-genome data [21]. Ding et al. [18] successfully developed SLIMP, a system capable of efficiently amplifying short gene sequences at concentrations as low as 1 aM. SLIMP is also able to quantify short gene sequences within a concentration range of 10 nM to 10 fM, offering high specificity, good selectivity, and feasibility for real-time testing [22]. These advancements demonstrate that ongoing optimizations and innovations in LAMP technology remain essential for addressing its current limitations, underscoring the need for continued efforts to improve the method’s capabilities.

Recombinase polymerase amplification (RPA) is an innovative isothermal amplification technique that has rapidly gained popularity since its introduction in 2006. It is distinguished by its simplicity, high sensitivity [23], and speed, allowing for the amplification of target sequences with as few as 1 to 10 copies in less than 20 min, even in the presence of inhibitors such as crude extracts or contaminants. RPA utilizes a strand-displacing DNA polymerase that efficiently elongates primers, leading to exponential amplification. It requires only two primers and an optional probe, making it a simple and efficient design. RPA employs recombinase enzymes along with accessory proteins for DNA unwinding and primer annealing. Key to the process are two proteins that replace the usual heat denaturation step in PCR: The Escherichia coli RecA recombinase and the single-strand DNA-binding protein (SSB). These proteins facilitate the formation of nucleoprotein filaments, allowing for efficient primer annealing and strand separation. A strand-displacing DNA polymerase, currently sourced from Staphylococcus aureus (SauDNA polymerase) in commercial kits, carries out the replication process. Additionally, accessory proteins like the T4 UvsY protein, a recombinase-loading factor, assist RecA in the nucleoprotein filament formation, further enhancing the efficiency of the RPA reaction. This combination of enzymes and cofactors supports the rapid and efficient amplification process, eliminating the need for thermal cycling [24]. This method is capable of amplifying double-stranded DNA, single-stranded DNA, methylated DNA, and cDNA [24]. Operating at temperatures between 22 and 45 °C [25], RPA does not require strict temperature control, making it an ideal option for diagnostic applications in resource-limited settings [23]. Its ability to function across a range of temperatures without compromising performance, along with its compatibility with multiplexing, makes RPA particularly attractive for point-of-care diagnostics [23]. Despite its advantages, RPA has some limitations. Currently, the kits are supplied by only one manufacturer, limiting flexibility and potentially increasing costs. Furthermore, post-amplification purification or digestion is often necessary to prevent interference in downstream detection. RPA can also be inhibited by high concentrations of genomic DNA, and non-specific detection methods, such as SYBR Green, cannot always distinguish amplicons from primer dimers. Additionally, conventional real-time PCR probes [26], such as TaqMan, are not compatible with this technique. While multiplexing is achievable with RPA, it requires the careful optimization of primer concentrations, and the lack of specific software for designing RPA primers complicates the optimization process [23]. Just like LAMP has demonstrated its efficacy in virus detection, recombinase polymerase amplification (RPA) has also shown promising developments. In recent developments, LG Liang and colleagues created an RPA assay for the simultaneous detection of SARS-CoV-2, as well as influenza A (H1N1 and H3N2) and influenza B, achieving a sensitivity of 100% and a specificity of 96.67% [27]. This highlights the potential of RPA for effective viral diagnostics. Similarly, Wongsamart et al. [28] introduced a multiplex RPA (mRPA) assay capable of detecting 20 high-risk and 14 low-risk HPV types in a single tube, with a low detection limit of 1000 copies for the L1 gene and 100 copies for the E6/E7 gene. The mRPA assay demonstrated 100% specificity, with diagnostic accuracy of 78% and sensitivity of 75% compared to commercial HPV tests like Cobas and REBA [28].

CRISPR-Cas (Clustered Regularly Interspaced Short Palindromic Repeat) systems and their associated proteins are currently at the forefront of research. The first CRISPR systems were detected 30 years ago in Escherichia coli during an analysis of the gene responsible for the isozyme conversion of alkaline phosphatase [29]. Since its discovery in 1987, the CRISPR-Cas system has revolutionized genome manipulation and evolved significantly [30]. Initially developed for treating genetic and infectious diseases, its applications have expanded to include fields such as molecular imaging [31]. In 2016, the CRISPR-Cas system was successfully integrated into molecular diagnostics, marking a turning point in disease detection. CRISPR-Cas-based diagnostic tools, known for their high sensitivity and specificity, sometimes outperform traditional PCR and do not require complex or expensive equipment, making them more accessible and cost-effective [12,32]. This innovation has the potential to radically transform diagnostics and global healthcare by effectively addressing the demand for rapid, accessible diagnostics. The CRISPR/Cas system consists of two main components: a guide RNA (gRNA) and a Cas nuclease, forming a ribonucleoprotein (RNP). The gRNA targets a specific sequence, and the Cas nuclease, upon binding to this target, performs cleavage (cis-cleavage). The CRISPR/Cas systems consist of several types of nucleases, each possessing unique characteristics that make them valuable for various applications. Among these nucleases, Cas12 and Cas13, which belong to class 2 CRISPR-Cas systems, are particularly useful in diagnostics due to their distinct ability to perform the collateral cleavage (trans-cleavage) of nucleic acids. Cas12 targets both single-stranded and double-stranded DNA, while Cas13 specifically targets single-stranded RNA. However, other CRISPR systems, such as Cas9 [33] and dCas9 [34], initially developed for genome editing, as well as Cas14 [35], have also been introduced into the field of molecular diagnostics. Although these proteins are primarily associated with genome editing, they have demonstrated potential in diagnostics, especially when used in conjunction with Cas12 and Cas13, which remain the most widely employed for viral infection detection. This non-specific cleavage, activated when the guide RNA-nuclease complex binds to a target sequence, triggers indiscriminate cleavage not only of the target but also of adjacent fluorescent reporters. These cleaved reporters enable the development of highly sensitive and specific diagnostic tests. Combining the sensitivity provided by isothermal amplification with the specificity of CRISPR/Cas systems has the potential to create a new decentralized, gold-standard diagnostic test that can be used directly at the point of care. These platforms could meet the WHO’s ASSURED criteria [8,36,37,38,39]. A study by Jiali et al. [40] compared the performance of PCR, qPCR, LAMP, and RPA-CRISPR/Cas12a technologies in molecular diagnostics, highlighting the strengths and limitations of each. Their findings demonstrated that LAMP exhibited the highest sensitivity among the evaluated methods, followed by qPCR and RPA-CRISPR/Cas12a. However, the two isothermal approaches, LAMP and RPA-CRISPR/Cas12a, offered significant advantages in operational simplicity, particularly due to their ability to function without thermocyclers, making them well suited for resource-limited settings [40].

Although significant research has focused on the use of CRISPR in diagnostics, recent bibliometric analyses highlight the growing interest in CRISPR-Cas technologies [31,41]. Various systematic reviews have indirectly emphasized the increasing importance of CRISPR-Cas technologies [42]. However, no specific bibliometric analysis or systematic review thoroughly examines the state of the art in viral infection detection using these technologies. In this context, this article adopts a bibliometric approach and utilizes scientific mapping to conduct a general and comprehensive review of the literature on the use of CRISPR-Cas for viral infection diagnostics between 2019 and 2024. The specific objectives of this study are as follows: (1) to fill this gap by providing a comprehensive overview based on data from Web of Science, SCOPUS, and PubMed, highlighting the advances and potential applications of these technologies in viral disease detection; (2) to illustrate the temporal trends in publications over the past five years; (3) to identify the main topics currently targeted; and (4) to analyze future research hotspots in this field in order to reveal potential knowledge gaps. The structure of this article is as follows: It begins with an overview of the current state and significance of CRISPR-Cas technologies in viral infection detection. Then, the methodology used for the bibliometric analysis and scientific mapping is detailed. The Results Section includes a performance and citation analysis, followed by scientific mapping. Finally, the Discussion Section addresses the main findings, and the article concludes with a summary of the study’s key discoveries.

## 2. Materials and Methods

### 2.1. Bibliometric Research

#### 2.1.1. Data Source

Bibliometric research was conducted according to the Preferred Reporting Items for Systematic Reviews and Meta-Analyses (PRISMA) statement [16]. Screening and data extraction were conducted independently by two individuals, OJ and LB.

The data were retrieved on 27 July 2024 from databases Web of Science (https://www.webofscience.com/), Scopus (https://www.scopus.com/), and PubMed (https://pubmed.ncbi.nlm.nih.gov/), all of which are renowned for their extensive coverage and reliability across various academic disciplines. Web of Science is recognized for its comprehensive coverage and robust citation data. Scopus, another subscription-based database, is widely used for its broad range of journals and depth in scientific and technical fields. PubMed, specifically oriented towards life sciences and biomedicine, offers free access to a vast collection of abstracts and articles, supported by strong citation data. The combined use of these three databases ensures the exhaustive coverage and increased relevance of results, providing a comprehensive overview of the available scientific literature on CRISPR-Cas systems and their application in the detection of viral infections. The search query was “(“CRISPR-Cas13*” OR “CRISPR/Cas13*” OR “Cas13*” OR “C2c2” OR “CRISPR-Cas12*” OR “CRISPR/Cas12*” OR “Cas12*” OR “CRISPR/Cpf1” OR “CRISPR-Cas9*” OR “CRISPR/Cas9*” OR “Cas9*” OR “CRISPR-Cas14*” OR “CRISPR/Cas14*” OR “Cas14*” OR “dCas9*” OR “Dead Cas9*”) AND (“virus*” OR “viral”) AND (“diagnosis” OR “detection” OR “detecting”)”. All identified articles were exported into a (BibTex) file from Web of Science and Scopus, while the results from PubMed were exported into a (txt) file.

The selected articles underwent a preliminary eligibility check based on their titles and abstracts, followed by a full-text review. Any disagreements were resolved by referring to a third reviewer.

#### 2.1.2. Inclusion and Exclusion Criteria

The selected articles had to meet the following inclusion criteria:

(i) All CRISPR-Cas Systems (Including Cas9, Cas12, Cas13, Cas14, and dCas);
(ii) Published Between 2019 and 2024;
(iii) Focused on Viral Infections, with Viruses As Major Keywords;
(iv) English;
(v) Open Access, Allowing Full Access to Complete Texts;
(vi) Only Original Research Articles Were Considered.

The study was limited to this specific period to ensure the relevance of recent and emerging data. Publications from 2024 were included in the analysis even though the year was not yet complete in order to capture the most recent developments.

**Figure 1:**
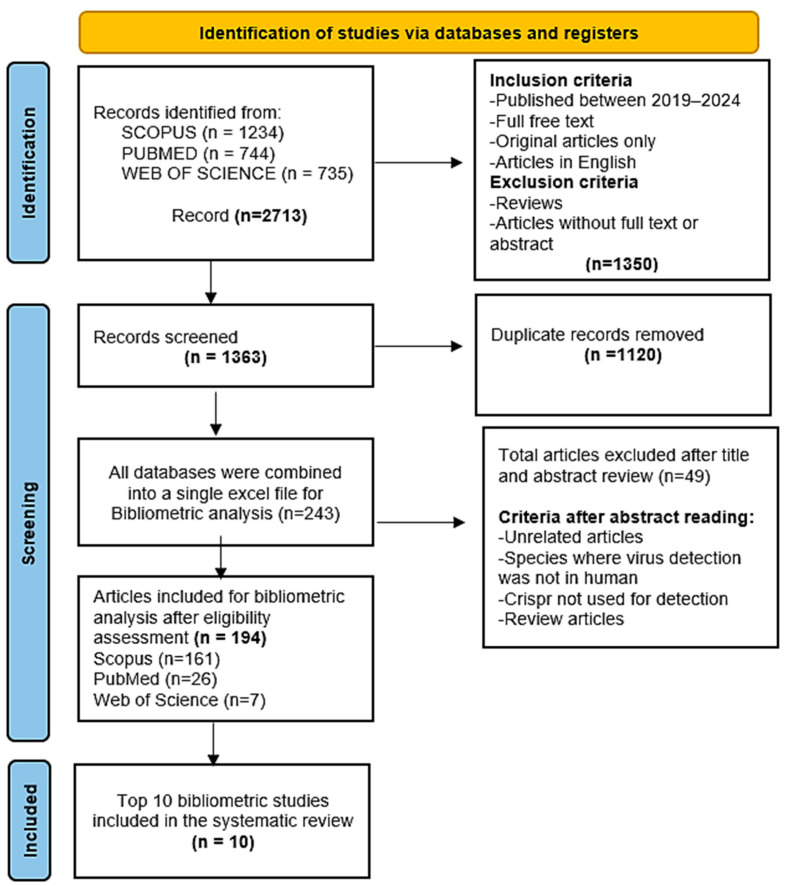
PRISMA flow diagram.

A systematic review was also conducted on the top 10 articles identified through the bibliometric analysis, as these studies provide a critical synthesis and comprehensive overview of existing research, which is essential for a thorough assessment of the current state of the art in this field. This rigorous selection ensures that the results accurately reflect the ongoing evolution and technological advances in the use of CRISPR-Cas systems for the detection of viral infections (Figure 1). From the eligible studies, the following data were extracted: authors and publication year; DOI; CRISPR protein/effector type and subtype; method name; target type; amplification method; assay time; sample type; detection steps; complementary technologies used; sensitivity (limit of detection); specificity; and aspects related to stability and portability, including the potential for point-of-care testing.

### 2.2. Bibliometric Processing

Bibliometric approaches, which have become essential for systematically analyzing bibliographic databases, offer a valuable quantitative method [43]. These techniques allow the classification and organization of scientific material based on criteria such as authors, institutions, and countries, thereby enhancing researchers’ understanding of various fields of study. In our study on CRISPR-based viral infection diagnostics, we utilized bibliometric tools to conduct a bibliometric analysis. We thoroughly explored articles from Scopus, Web of Science, and PubMed databases using methods such as citation and publication analysis, an examination of authors and co-authors, and co-occurrence and keyword analysis. The analysis was performed using the R 4.3.1 package “bibliometrix” (http://www.bibliometrix.org) [44] on 6 July 2024, and VOSviewer [45], which are powerful tools for quantitative research in bibliometrics and scientometrics. The R package “bibliometrix” provides a set of tools for the descriptive analysis of bibliographic data, while VOSviewer allows for the construction and visualization of co-authorship and co-occurrence networks.

The web interface “biblioshiny”, integrated into the bibliometrix package, was used to show the evolution of scientific production on CRISPR-Cas, identify the most relevant sources, analyze thematic trends, and visualize collaboration networks. VOSviewer v1.6.17 was employed to perform network and cluster analyses, focusing particularly on keyword co-occurrence. These tools enabled a comprehensive overview of research trends and collaborations in the field of CRISPR-based diagnostics, highlighting major contributions and key connections within the scientific community.

## 3. Results

### 3.1. Publication Outputs Analysis from 2019 to 2024

**Figure 2:**
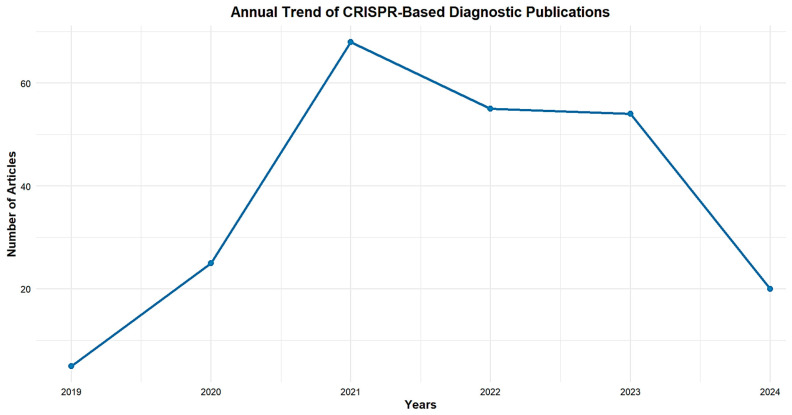
The number of publications of the related documents from 2019 to 2024.

Based on the retrieved records (Figure 2), publications on the CRISPR-Cas system for viral infection diagnosis were focused on. Therefore, documents between 2019 and 2024 were examined to assess the relevant research output. Overall, a total of 194 publications records were retrieved from the three databases (Scopus, Web of Science, and PubMed) using the search query described in the methodology. The number of publications was only 5 papers in 2019, suggesting that research in this field was still in its early stages, and it peaked at 68 publications in 2021, suggesting a growing recognition of CRISPR-Cas’s potential for viral diagnostics, including its accuracy, speed, and suitability for usage in decentralized environments. Despite a slight decline to 54 articles in 2022, the number of publications remained high, indicating stability in the scientific community’s interest, followed by 53 papers in 2023.

However, in 2024, there has been a more noticeable decline, with only 20 papers published so far.

### 3.2. List of the Most Common Viruses for Which CRISPR-Cas-Based Diagnostic Tests Have Been Developed

**Figure 3:**
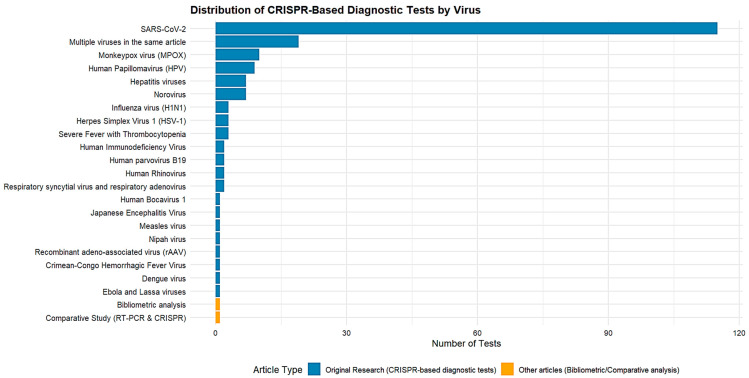
List of the most common viruses for which CRISPR-Cas-based diagnostic tests have been developed.

Figure 3 illustrates the number of diagnostic tests developed for different viruses using the CRISPR-Cas systems. SARS-CoV-2 dominates with 115 tests, followed by articles addressing several viruses in a single study (19 tests). Monkeypox virus (MPOX) and human papillomavirus (HPV) followed with 10 and 9 tests, respectively. Other viruses, such as hepatitis viruses, norovirus, and H1N1 influenza virus, present between 3 and 7 tests. Finally, less-studied viruses such as measles, Ebola, and Japanese encephalitis show a limited number of tests. This predominance of respiratory viruses in CRISPR-based diagnostics is not solely due to differences in the intrinsic efficiency of CRISPR detection systems but is primarily influenced by practical and clinical factors. Respiratory viruses are typically detected in easily accessible and non-invasive samples such as nasal swabs, saliva, or sputum. These sample types are highly compatible with rapid, point-of-care diagnostics, a setting where CRISPR technologies have been widely applied. In contrast, blood-borne viruses require venous blood collection, which involves trained personnel and is not easily adaptable to home-based testing. Furthermore, blood samples often require plasma separation, nucleic acid extraction, and the elimination of enzymatic inhibitors such as hemoglobin or proteases, which introduce operational barriers and complexity to CRISPR workflows [32,46].

Nevertheless, this does not imply that CRISPR diagnostics for blood-borne viruses are absent; on the contrary, such studies are ongoing but typically involve multi-step protocols. The current research focus is largely directed towards simplifying these workflows into single-step, amplification-free formats, which are more suitable for commercialization and even at-home use. This explains the growing interest in integrating advanced platforms such as microfluidics, lab-on-a-chip systems, and biosensors to minimize user manipulation; automating sample preparation from whole blood, ideally even from fingerstick-derived capillary samples; and enabling fully portable and self-contained diagnostic tools.

### 3.3. Analysis of Main Journals

**Figure 4:**
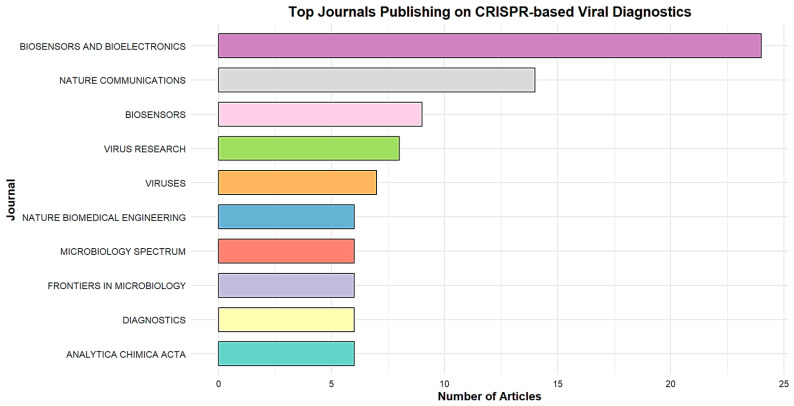
Top 10 productive journals on CRISPR-Cas systems for viral infection detection from 2019 to 2024.

Figure 4 highlights the 10 most prolific journals publishing research on CRISPR-Cas systems applied to the diagnosis of viral infections. *Biosensors and Bioelectronics*, with 24 publications, and *Nature Communications*, with 14 publications, stand out for their outstanding contributions to the dissemination of research in this field. Together, these two journals reflect the excellence and significant impact of the work published, drawing the attention of the scientific community to recent advances in CRISPR technology. *Biosensors* and *Virus Research* also stand out, with eight and seven publications, respectively. These figures underscore the interdisciplinary nature of this research, which combines in-depth studies of viruses and detection devices.

Finally, several other journals, such as *Analytica Chimica Acta*, *Diagnostics*, *Frontiers in Microbiology*, *Microbiology Spectrum*, and *Nature Biomedical Engineering*, each with five publications, contribute a diversity of perspectives. These journals cover a wide range of disciplines, from analytical chemistry to microbiology to biomedical engineering, illustrating the richness and diversity of approaches in this field.

### 3.4. Most-Contributing Authors, Countries, and Institutions

The authors, nations, and organizations that have made the greatest contributions are frequently those that have published the highest number of articles in a certain field or those whose works have had the highest number of citations. Finding the most influential authors in the field can yield invaluable information about the state of research in a particular field. Additionally, identifying the authors who have contributed the most may help identify research networks and collaborative efforts.

**Figure 5:**
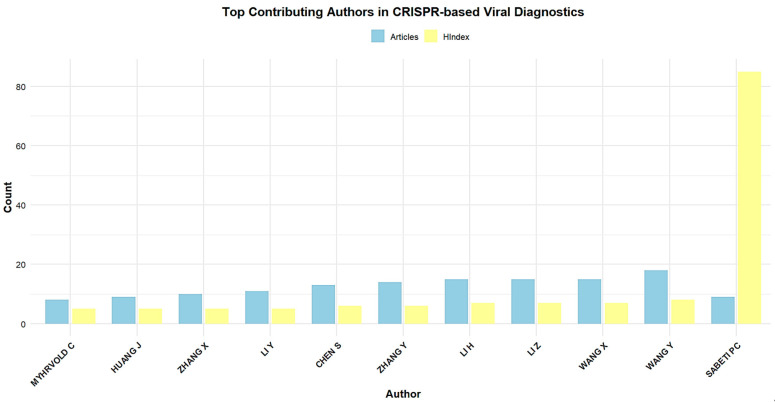
Top 10 authors who published on CRISPR-Cas systems for viral infection detection from 2019 to 2024.

Figure 5 demonstrates the top 10 most prolific authors in the field of CRISPR-Cas systems for viral infection diagnosis from 2019 to 2024. Leading the group is Wang Y with 20 publications and an H-index of 8, reflecting both high productivity and a growing impact in the field. Li H and Wang X follow closely with 16 and 15 articles, respectively, demonstrating substantial contributions, although their H-indices (6 and 7) indicate a slightly lower citation impact compared to others. Notably, authors such as Li Z and Zhang Y have produced 15 and 12 articles, respectively, with Li Z showing a significantly higher H-index of 11, suggesting stronger citation influence. Other authors like Chen S and Li Y have made considerable contributions as well, with 12 and 10 articles, each maintaining solid H-indices of 7. Sabeti PC stands out with an H-index of 78, despite having authored fewer articles (8), indicating a substantial and well-established influence in the field. Myhrvold C also has a high H-index of 19 with eight publications, underscoring the significant impact of their research. This table showcases both the quantity of research output and the relative impact of these authors, with Wang Y, Li Z, and Sabeti PC demonstrating particularly strong influence in CRISPR-Cas-based viral infection diagnostics.

**Figure 6:**
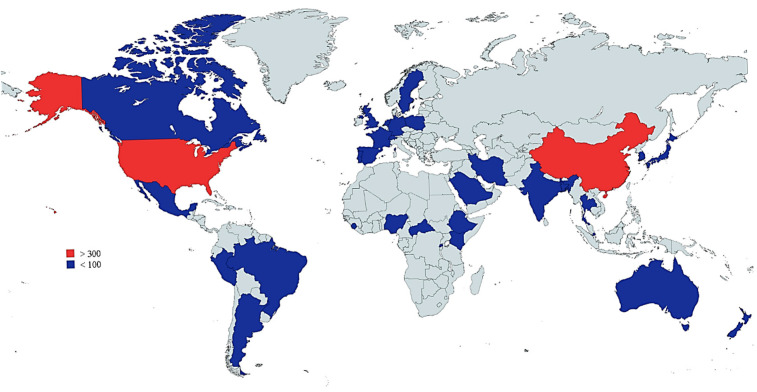
Global scientific production on CRISPR-Cas systems for viral infection detection from 2019 to 2024.

Figure 6 displays the number of documents produced by different countries (in terms of authors) that have contributed to research in viral infection detection using the CRISPR-Cas system. With 450 articles, the United States dominates the field, demonstrating its leadership in research and development in this area. China follows closely behind with 421 articles, indicating robust research activity as well.

With a lower number of documents, India (52), Canada (40), South Korea (42), and Japan (36) contributed to the enrichment of the fields.

**Figure 7:**
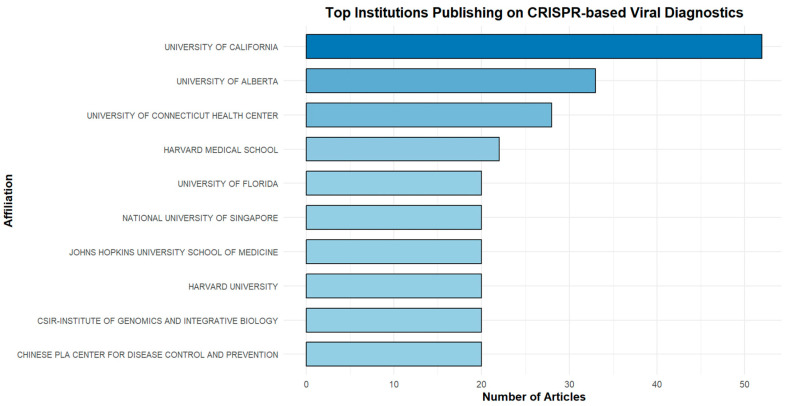
Top 10 productive institutions on CRISPR-Cas systems for viral infection detection from 2019 to 2024.

Figure 7 shows the top 10 relevant affiliations. The University of California is the most prolific university with 53 articles. This indicatively demonstrates the extensive range of studies conducted in this particular area at this educational institution.

The University of Alberta ranks second with 31 papers, while the University of Connecticut Health Centre follows closely with 27 papers, underscoring their substantial contribution to CRISPR-Cas research.

### 3.5. Co-Occurrence Keywords and Co-Authorship of Authors

#### 3.5.1. Co-Occurrence Keywords

**Figure 8:**
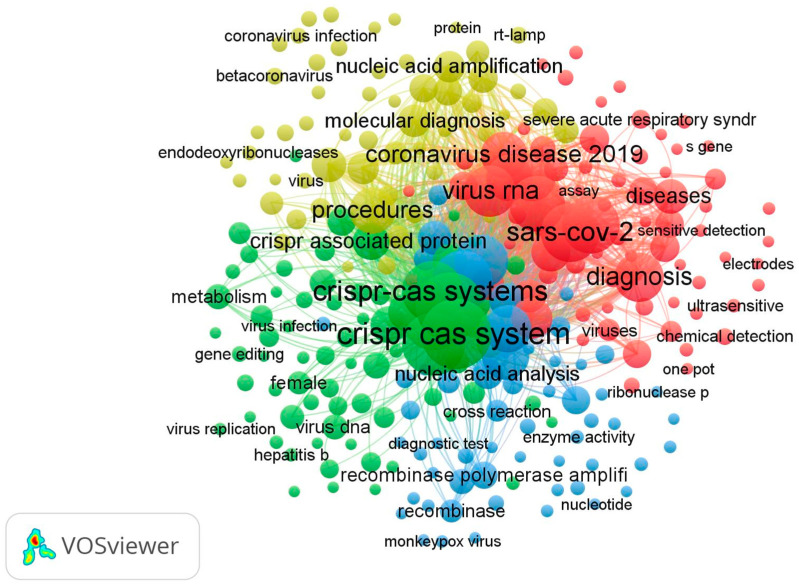
Keywords co-occurrence (*n* ≥ 5) clusters of CRISPR-Cas systems from 2019 to 2024 for viral infection detection, visualized by VOSviewer.

VOSviewer was assessed only on the Scopus database, which contains the most publications about CRISPR-Cas systems for viral infection detection from 2019 to 2024 in comparison with the Web of Science and PubMed databases. In Figure 8, 4 main clusters of research were identified from a total of 194 (with a minimum number of occurrences of a keyword, *n* ≥ 5). The keyword “Crispr Cas system” had the highest frequency at 140, and it had a strong link with “Genetics”. The overall keywords reveal four clusters related to CRISPR-Cas systems for viral infection detection.

The green cluster represents, in general, the mechanism of action of the Crispr Cas system in genetics using the occurrence of various keywords, such as “RNA extraction”, “Gene amplification”, “DNA template”, “Gene editing”, “Gene expression”, “Crispr Cas system”.

The blue cluster research theme is based on the sensitivity and specificity of the Crispr Cas system in virus detection. It is characterized by the presence of the keywords “Diagnostic accuracy”, “Limits of detection”, “Monkey pox virus”, “Influenza virus”, and “SARS-CoV-2”.

Red cluster research was specific to the diagnosis of SARS-CoV-2, which contains the following keywords: “COVID-19”, “severe acute respiratory syndrome”, “Virus RNA”, “Polymerase Chain Reaction”, and “Reverse transcription polymerase”.

Finally, the yellow cluster focuses on the procedure of molecular diagnosis; this cluster contains keywords such as “Molecular diagnostic technique”, “Real time reverse transcription”, “Isolation and purification”, “Nucleic acid amplification”, and “Open reading frame”.

#### 3.5.2. Co-Authorship of Authors

**Figure 9:**
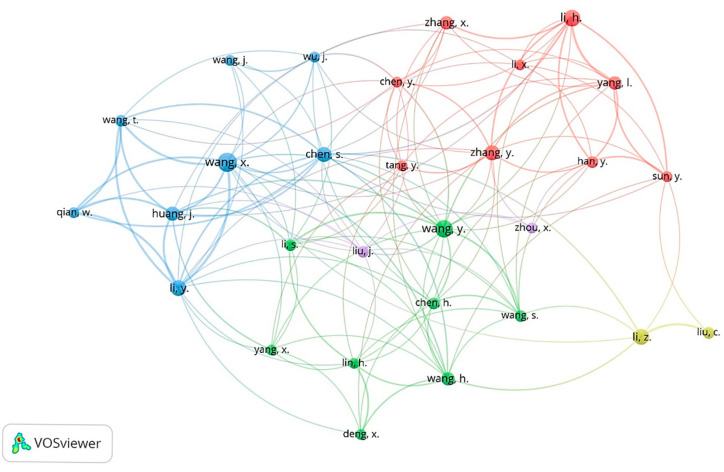
Authors’ collaboration network.

A total of 1125 authors produced the 194 publications present in the dataset. In Figure 9, only authors who have written a minimum of five publications were retained. The co-authorship network analysis performed by VOSviewer allowed us to identify five clusters with a total of 29 authors. Wang, X had the highest number of publications and was linked with all the clusters, revealing a dynamic collaboration with other authors. Similarly, Chen, S also carried out collaboration, as observed via all the clusters, but had a lower number of publications (*n* = 9).

#### 3.5.3. Most Cited Articles in CRISPR-Based Viral Detection

**Figure 10:**
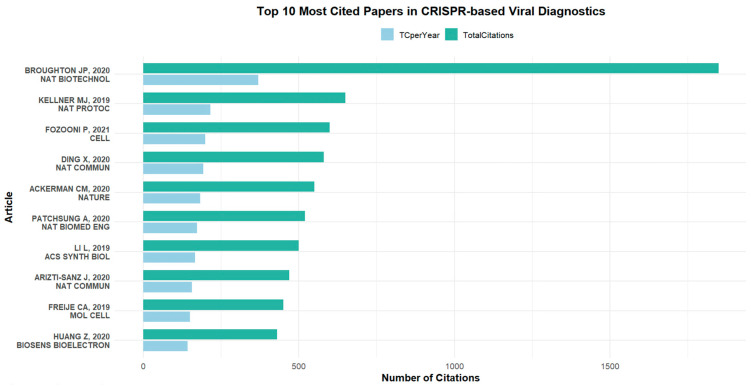
Citation counts of the top 10 most cited articles in the field of CRISPR-based viral detection [47,48,49,50,51,52,53,54,55,56].

To better understand the foundational studies that have shaped research in CRISPR-based viral detection, we identified and summarized the most highly cited publications in this field. Table 1 presents the top 10 most cited articles, including pioneering works that introduced or optimized CRISPR-based diagnostic platforms. To provide a clearer overview of their impact, Figure 10 illustrates the citation counts for these articles. Together, these elements highlight the landmark contributions that have driven innovation and served as key references for subsequent research.

### 3.6. Mechanisms of Cas12 and Cas13 Enzymes in Molecular Detection

As summarized in Table 2, the diagnostic systems described in the most influential studies overwhelmingly rely on the Cas12 and Cas13 proteins.

**Figure 11:**
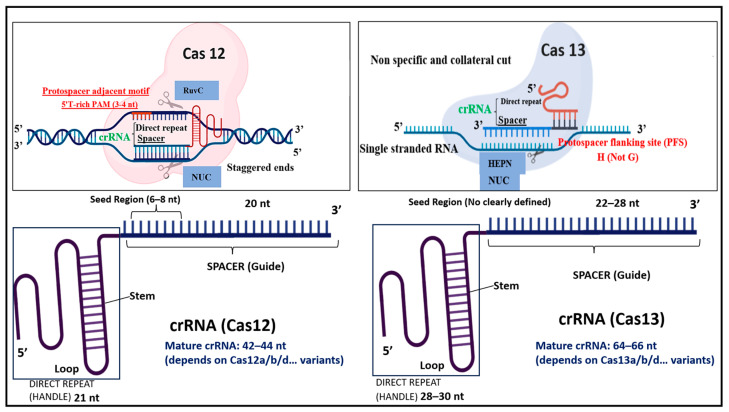
Schematic illustration of the molecular mechanisms of CRISPR/Cas12 and CRISPR/Cas13 systems, highlighting their crRNA structure, target interaction, and cleavage behavior. Cas12 proteins bind to double-stranded DNA near a thymine-rich PAM sequence (e.g., TTN or TTTN), initiating an R-loop formation via crRNA–DNA hybridization. Once bound, Cas12 employs its RuvC domain to cleave both DNA strands, producing staggered cuts. In contrast, Cas13 targets single-stranded RNA sequences in a PAM-independent manner, requiring instead a protospacer flanking site (PFS), typically a non-G nucleotide. Upon target recognition, Cas13 becomes activated and initiates both the specific cleavage of the target RNA and non-specific collateral cleavage of surrounding ssRNA molecules. The crRNAs guiding Cas12 and Cas13 differ in size and structural organization, with Cas12 utilizing a mature crRNA of 42–44 nt (including a 21 nt direct repeat), while Cas13 uses a longer crRNA of 64–66 nt with a 28–30 nt direct repeat. The seed region in Cas12 is well defined (6–8 nt), while Cas13 lacks a strictly conserved seed sequence.

Cas12 proteins, members of the class 2 type V CRISPR effectors, have emerged as central tools in molecular diagnostics, particularly for the detection of microbial and viral pathogens [57]. This family encompasses a diverse array of subtypes, including Cas12a, Cas12b, Cas12c, Cas12e, Cas12f, Cas12g, Cas12i, Cas12j, Cas12k, and Cas12m, which are classified based on their amino acid length, PAM requirements, cleavage activity, and target nucleic acid substrate [58]. Among them, Cas12a (formerly Cpf1) and Cas12b (formerly C2c1) are the most widely used in diagnostic applications. These nucleases, typically composed of 1200 to 1500 amino acids, recognize thymine-rich PAM sequences (e.g., TTN) and cleave double-stranded DNA (dsDNA) to generate staggered 5′ overhangs [58]. Unlike Cas9, which requires both a crRNA and tracrRNA, Cas12a and Cas12b operate with a single crRNA, simplifying the guide RNA design and enhancing adaptability in point-of-care settings [59]. A hallmark of Cas12 enzymes is their collateral (trans-) cleavage activity upon specific target recognition (Table 3); the activated effector nonspecifically cleaves nearby single-stranded DNA (ssDNA), a property that has been successfully harnessed for fluorescence-based and lateral flow diagnostics using labeled reporter probes [59]. Cas12 protein contains the RuvC and nuclease lobe (NUC) domains for cleavage activity (Figure 11) [60]. Upon recognition of its target, Cas12 initiates the formation of an R-loop structure, characterized by the hybridization between the CRISPR RNA (crRNA) and the complementary strand of the target DNA [57]. This process begins with the base pairing of approximately 17 bp (min) between the crRNA and the target sequence, which facilitates the displacement of the non-complementary DNA strand and stabilizes the R-loop. Once this structure is established, the RuvC catalytic domain of Cas12 becomes activated and cleaves the non-target DNA strand, a process that is PAM-dependent, requiring the presence of a thymine-rich protospacer adjacent motif (PAM). While the role of the RuvC domain in non-target strand cleavage is well characterized, its precise involvement in the cleavage of the target DNA strand remains insufficiently understood and is still under investigation [57]. The first Cas12-based diagnostic platform was introduced in 2018 and relied on the coupling of Cas12a with a fluorophore-quencher (FQ)-labeled single-stranded DNA reporter molecule [36]. Upon the recognition and binding of the target DNA, Cas12a formed a ternary complex with the crRNA and the target, which triggered its characteristic trans-cleavage activity. This activation enabled Cas12a to nonspecifically cleave the ssDNA FQ reporter, thereby releasing the fluorescent signal. Using this approach, researchers achieved the rapid and specific detection of two double-stranded DNA viruses, human papillomavirus type 16 (HPV16) and type 18 (HPV18), with results obtained in less than one hour [36]. Recently, Cas12f (previously known as Cas14) has attracted significant attention due to its ultracompact size (approximately 400–700 amino acids) and unique functional properties. Unlike other Cas12 subtypes that function as single effectors, Cas12f operates as a homodimer to bind and cleave its target DNA. Notably, it does not require a PAM sequence and can target ssDNA, offering new possibilities for integration into miniaturized or field-deployable diagnostic platforms. These characteristics make Cas12f a promising candidate for portable and cost-effective diagnostic systems, particularly in resource-limited environments [57,61].

Cas13 is a type VI RNA-guided RNA-targeting enzyme, a member of the enzyme family that includes Cas13a, Cas13b, Cas13c, and Cas13d [62,63]. Cas13 specifically cleaves ssRNA and not dsRNA, and its unique features have been used in diagnoses [59]. The RNA cleavage is mediated by two higher eukaryote and prokaryote nucleotide-binding (HEPN) domains (Figure 11), which are commonly found in ssRNA specific endoribonucleases, such as csm6 [9,64]. Similarly to Cas12, Cas13 proteins exhibit a dual-mode activity involving both the cis-targeted cleavage and trans-cleavage of non-target molecules (Table 3) [57]. Upon recognition and binding to their complementary single-stranded RNA (ssRNA) targets, Cas13 enzymes undergo a conformational change that activates their collateral cleavage activity, enabling the indiscriminate degradation of surrounding non-target ssRNA molecules. This unique property, comprising the trans-cleavage activity of Cas13, forms the molecular foundation of many CRISPR-Cas13-based diagnostic platforms. An early and notable example is the SHERLOCK platform (Specific High-Sensitivity Enzymatic Reporter Unlocking), which was first introduced in 2017 and demonstrated the potential of Cas13 for highly sensitive RNA detection [65]. Cas13a (previously called C2c2), one of the most widely used subtypes, is guided by a single crRNA, without the need for a tracrRNA, simplifying its use in in vitro diagnostic settings. The enzyme is composed of an NUC lobe and two conserved HEPN (higher eukaryote and prokaryote nucleotide-binding) RNase domains that are responsible for both sequence-specific and collateral RNA cleavage. Cas13a recognizes target sequences that are typically 22–28 nucleotides in length [65], and activation is influenced by a protospacer flanking site (PFS) at the 3′ end of the target, which exhibits a preference for adenosine (A), uracil (U), or cytosine (C), a feature that distinguishes it from the PAM requirement observed in Cas9 or Cas12 systems [57]. Upon successful target recognition, the enzyme cleaves preferentially near the uracil-rich regions of the ssRNA. Cas13b, another subtype of the Cas13 family, has shown greater target specificity compared to Cas13a [57]. This subtype recognizes a PFS at the 5′ end with a preference for A, U, or G, and sometimes incorporates a PAM-like motif (NAN or NNA) at the 3′ end [66]. Cas13b is also guided by a mature crRNA, and the CRISPR/Cas13b complex is capable of inducing conformational changes in the target RNA upon binding, which in turn activates collateral RNase activity [57]. While the full mechanistic details of Cas13b remain under investigation, recent studies have also explored its potential for RNA editing applications [67], expanding its utility beyond detection to include functional transcriptome engineering. A key practical distinction is that Cas13 requires an additional in vitro transcription step using T7 RNA polymerase to convert amplified DNA into RNA, whereas Cas12 can directly act on DNA targets [68].

The continued exploration and characterization of Cas12 and Cas13 subtypes are essential to expand the toolkit of CRISPR-based diagnostics, especially for the detection of emerging and re-emerging infectious diseases, including viral pathogens.

### 3.7. Visual Overview of CRISPR-Cas Diagnostic Platforms and Detection Strategies

**Figure 12:**
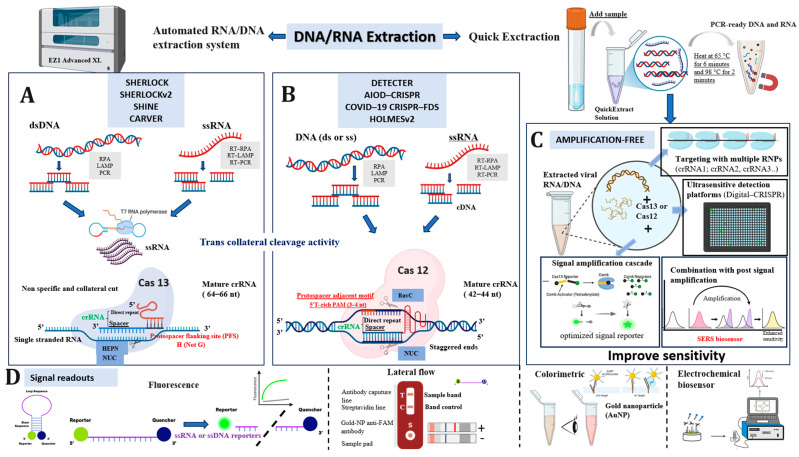
Simplified illustration of CRISPR-Cas12 and Cas13 detection workflows and signal readout strategies for nucleic acid diagnostics. (**A**) Amplification-dependent detection using CRISPR-Cas13 systems. DNA or RNA targets are first amplified (via RPA, LAMP, or PCR), then transcribed (for dsDNA) into ssRNA before Cas13-mediated detection. Cas13 exhibits trans collateral cleavage activity upon target recognition. (**B**) Amplification-dependent detection using CRISPR-Cas12 systems. DNA or cDNA is amplified and detected through Cas12-mediated cleavage activity. Cas12 recognizes target DNA via a specific PAM sequence and exhibits staggered cleavage. (**C**) Amplification-free strategies to enhance sensitivity. These include direct detection of viral nucleic acids by Cas12 or Cas13 without prior amplification, the use of multiple crRNAs (RNP multiplexing), ultrasensitive platforms (e.g., Digital CRISPR), signal amplification cascades, and post-signal enhancement techniques such as SERS biosensors. (**D**) CRISPR-based signal readout methods. The figure illustrates four commonly used detection strategies in CRISPR diagnostics: Fluorescence readout target recognition induces collateral cleavage and fluorescence signal; the green and grey curves represent the presence and absence of target, respectively. Lateral flow assays a visible band at the test line (T) indicates a positive result. Colorimetric detection AuNP aggregation produces a visible color change from yellow to red. Electrochemical biosensor signal detection is based on voltage/current changes in response to target recognition.

Figure 12 provides a comprehensive schematic representation of the general diagnostic workflow employed in the top 10 most-cited articles from our bibliometric analysis, illustrating the diversity and modularity of CRISPR-based detection systems. The process begins with nucleic acid extraction, which may be performed using an automated extraction system (for example, EZ1 Advanced XL) or through rapid, heat-based protocols that enable direct sample processing in less than 10 min. Following extraction, the viral genetic material, either RNA or DNA, is subjected to amplification. Depending on the viral genome type, different amplification strategies are used; RT-PCR or isothermal methods such as RT-RPA and RT-LAMP are applied for RNA viruses, while RPA, LAMP, or PCR are preferred for DNA viruses. The selection of the CRISPR effector enzyme is a crucial step. Cas13, which possesses RNase activity, is strictly specific to single-stranded RNA (ssRNA) targets. Consequently, for RNA viruses, amplification yields cDNA, which is then transcribed into RNA via T7 RNA polymerase to enable Cas13 recognition. These systems rely on the collateral cleavage activity of activated Cas13, which, upon target binding, nonspecifically cleaves labeled RNA reporters to generate a detectable signal.

In contrast, Cas12 exhibits both DNase activity and a preference for double-stranded DNA (ds or ssDNA) targets, although it can also recognize single-stranded DNA (ssRNA). Therefore, when using Cas12 (e.g., in DETECTR, AIOD-CRISPR, HOLMESv2, or COVID-CRISPR-FDS), amplification products from both RNA and DNA viruses are typically in DNA form, eliminating the need for a T7 transcription step. Detection outputs across both Cas systems are typically visualized through several signal readout methods, including fluorescence, lateral flow assays, colorimetric detection, and electrochemical biosensors. Each approach can be adapted depending on the setting (e.g., laboratory vs. field diagnostics).

One of the top-cited studies identified in our bibliometric analysis introduced an amplification-free CRISPR-based detection strategy, representing a significant advancement in terms of speed, workflow simplicity, and potential for deployment in point-of-care (POC) settings. By eliminating the nucleic acid preamplification step, this approach not only reduces the overall detection time but also minimizes critical drawbacks commonly associated with traditional amplification methods, such as nonspecific amplification, primer dimer formation, and cross-interference [69]. Furthermore, classical two-step workflows (amplification followed by detection) increase the risk of aerosol contamination and require additional handling [70]. In contrast, amplification-free strategies enable single-tube reactions, reducing contamination risk and making the system more suitable for decentralized or field-based diagnostics [70]. Nevertheless, the absence of target amplification presents a major challenge in achieving adequate analytical sensitivity. To address this, several optimization strategies have been developed. These include the rational design of CRISPR RNA guides (crRNAs) to enhance specificity and trans-cleavage activity, as well as the use of multiple synergistic crRNAs targeting different regions of the same viral sequence to boost the signal-to-noise ratio [49,70,71]. Enhanced signal generation has also been achieved through the development of high-efficiency fluorescent reporters, the incorporation of RNA aptamers with dye-binding capabilities, and the use of nanomaterials such as gold nanoparticles (AuNPs) to harness metal-enhanced fluorescence [49,72]. Additionally, the adoption of digital detection platforms such as Digital-CRISPR enables the compartmentalization of the reaction into nanoliter droplets, increasing the local concentration of the target and pushing the limit of detection (LOD) to picomolar or even single-molecule levels [73]. Furthermore, kinetic modeling studies based on Michaelis–Menten parameters have shown that the catalytic efficiency (e.g., k_cat, K_M) of the Cas enzyme complex is influenced by crRNA structure, reporter design, and target sequence composition [74]. This underscores the importance of selecting the most catalytically efficient combinations. Additional improvements have also emerged through the integration of post-cleavage amplification circuits, signal transducers, and alternative readout modalities beyond traditional fluorescence, including electrochemical sensors [75], surface-enhanced Raman spectroscopy (SERS) [76], and nanopore-based technologies [77].

## 4. Discussion

The impact of the COVID-19 pandemic on global society has underscored the critical importance of rapid and accurate diagnostic tools. Since late 2019, the term “positive” has taken on significant weight due to the highly infectious nature of the SARS-CoV-2 virus. The emergence of infectious diseases such as SARS-CoV-2 has highlighted the urgent need for efficient, accessible, and resource-adapted detection methods, particularly in low-resource settings [14,78]. Studies such as those by Kwon and Shin have emphasized the importance of rapid diagnostic systems in containing the spread of highly infectious viruses [59]. One of the technologies that gained significant popularity during this crisis is the CRISPR-Cas system, including Cas9, Cas12, Cas13, and Cas14, due to its ability to deliver real-time results with remarkable accuracy. Although the initial applications of CRISPR in diagnostics began as early as 2016, a major breakthrough in this field was marked by the pioneering work of Gootenberg et al. in 2017 [65], when they introduced the SHERLOCK (Specific High-Sensitivity Enzymatic Reporter UnLOCKing) assay. This innovative technique, which leverages the collateral activity of CRISPR-Cas13a combined with isothermal amplification, enabled the rapid and precise detection of specific Zika and dengue virus strains. This success paved the way for the rapid expansion of CRISPR-Cas-based diagnostics, firmly establishing this technology as a powerful tool for virus detection [65]. CRISPR-Cas systems are distinguished by their speed and specificity, making them particularly suitable for point-of-care testing (POCT), where rapid results are crucial [37]. Their ability to be effectively deployed in low-resource settings, as well as in temporary facilities, makes them valuable for a wide range of clinical contexts, from pharmacies to patients’ homes [79]. Being easy to use, sensitive, specific, cost-effective, and reliable, they do not require highly skilled personnel, making them an ideal solution for accessible and rapid diagnostics. Their versatility in targeting various pathogens and their integration into high-throughput screening platforms further solidify their role in combating pandemics and emerging diseases [80]. CRISPR-Cas systems represent an indispensable tool in modern molecular diagnostics, effectively addressing current public health challenges [78].

Between 2019 and 2024, research on CRISPR-Cas systems experienced exponential growth, reflecting an increasing interest in their application for the detection of infectious diseases [40]. These technologies have emerged as promising tools due to their ability to deliver rapid, specific, and sensitive diagnostics, particularly in the context of viral infections. Our bibliometric analysis focused on this dynamic by examining articles reporting the development or validation of CRISPR-Cas-based diagnostic tests exclusively targeting viral infections. By exploring three major databases, a significant number of tests were identified, with 115 specifically designed for the detection of SARS-CoV-2. These collaborative efforts not only highlight the global response to pressing health challenges but also underscore the profound impact of SARS-CoV-2 on research priorities in molecular diagnostics [81]. Beyond SARS-CoV-2, other viruses have also garnered increasing interest, notably the monkeypox virus (Mpox). This zoonotic disease, caused by the monkeypox virus, has been marked by recent outbreaks with heightened global spread. However, diagnosing this virus remains a challenge due to its often-ambiguous clinical presentation and the need for highly specific tests [82]. CRISPR-Cas technologies, due to their speed and sensitivity, offer an innovative solution to these limitations. For instance, the study by Zhao et al. [83] demonstrated a one-tube method combining RPA and Cas12a, enabling the detection of minute amounts of viral DNA down to a single copy per reaction in just 30 min. This type of innovation highlights the capability of CRISPR-Cas diagnostics to address the urgent need for rapid and accurate testing in various contexts, ranging from global pandemics like COVID-19 to emerging diseases such as Mpox [83,84]. Moreover, our study highlights a diverse spectrum of CRISPR-associated proteins used in the literature. Cas12a and Cas13a emerge as the most prevalent, accounting for two-thirds of the articles. While these proteins dominate, other CRISPR-associated proteins, such as Cas9, Cas14, and dCas, are also being explored, albeit to a lesser extent. Their presence in the literature reflects the ongoing exploration and diversification of CRISPR-based technologies, thereby enriching our understanding of the utilization and prevalence of the CRISPR-Cas system.

Recent advancements in molecular diagnostics provide promising solutions for detecting viral infections in resource-limited settings. An optimal diagnostic test must combine precision and sensitivity to identify the pathogen while being accessible, easily transportable, and capable of differentiating various variants. This aligns with the ASSURED criteria (Affordable, Sensitive, Specific, User-friendly, Rapid, Equipment-free, and Deliverable to end-users) defined by the WHO [85]. The CRISPR-Cas13 system, in particular, has proven to be promising for the rapid detection of SARS-CoV-2 in clinical settings [86]. Although several CRISPR-based tests have been developed, only a few have advanced to clinical use, highlighting the need for broader implementation [87]. Significant research efforts have been dedicated to integrating CRISPR-Cas-based diagnostics with various signal detection methods, such as lateral flow immunochromatography (LFA), fluorescence, electrochemistry, microfluidics, microarrays, and electrochemical biosensors. These approaches aim to advance point-of-care testing (POCT) by utilizing portable devices or low-cost readers while delivering rapid and accurate results [50,88]. Among these methods, LFA stands out for its simplicity, rapid processing time, and low cost, making it a powerful tool for CRISPR-Cas-based diagnostics, particularly in settings without access to complex laboratory infrastructure. Technologies such as SHERLOCKv2 and NASBACC, for instance, utilize gold nanoparticles for clear colorimetric visualizations through LFA assays, enabling high sensitivity with short analysis times [89]. Furthermore, the integration of microfluidics and advanced technologies, such as protein engineering and artificial intelligence, further enhances the portability and performance of CRISPR-Cas diagnostics for POCT. For instance, a recent advancement in protein engineering has improved the sensitivity of Cas13 in amplification-free detection systems. By fusing an RNA-binding domain (RBD) to LwaCas13a, enzymatic activity was significantly enhanced, achieving 518% higher fluorescence than the wild-type version for specific targets. This approach enabled a sensitivity of 0.6 copies/μL of synthetic RNA and 12 copies/μL in clinical samples within just 30 min, without preamplification [89]. However, further clinical validation is needed to expand these applications. This engineering strategy could also be adapted to other Cas13 variants, including thermostable variants such as TccCas13a and HheCas13a, by tailoring the RBD domains to their thermophilic properties. These innovations, coupled with continuous improvements in sensitivity, selectivity, and chemical stability, significantly broaden the applicability of CRISPR-Cas systems for the rapid and efficient detection of various pathogens [90,91].

One of the main challenges in validating the test in the laboratory lies in off-target effects, where Cas proteins may inadvertently cleave non-target DNA or RNA sequences. This phenomenon, often caused by off-target recognition by the guide RNA (gRNA), can lead to collateral cleavage and false-positive results. To address this limitation, optimization strategies for gRNA sequences and Cas enzymes have been extensively explored to enhance the specificity of CRISPR/Cas systems [86]. In parallel, sensitivity in diagnostics remains another major challenge. Although techniques such as SHERLOCK and HOLMES combine nucleic acid amplification with CRISPR detection, these approaches rely on separate steps, increasing the risk of cross-contamination and complicating procedures [92]. The challenge lies in developing integrated systems capable of amplifying and detecting nucleic acids in a single step while maintaining high sensitivity. Scaling up to practical use and assuming the tests are validated in the laboratory, two major challenges limit the use of CRISPR-based diagnostics for POC and at-home applications. First, sample processing requires lengthy protocols and, in most cases, relies on specialized instrumentation [9]. Secondly, the reaction components must be stored and transported at ultra-low temperatures. The ultimate goal is to develop a one-step test that can be widely deployed without relying on a cold chain supply [93]. Moreover, the development of a POC test that meets all ASSURED criteria does not necessarily guarantee its usability in home environments. The test must be simple and easy to perform by an untrained individual, with minimal risk of generating erroneous results [93]. Furthermore, CRISPR-based diagnostics face commercialization challenges due to the limited standardization of protocols and logistical constraints related to the required infrastructure. Despite these challenges, ongoing efforts to optimize CRISPR techniques, enhance enzyme specificity, and reduce operational steps hold promise for overcoming these limitations and promoting their large-scale adoption in diagnostics. Considering these challenges, the Lucira Check It COVID-19 test kit (72) is currently the only FDA-approved molecular diagnostic test kit for home use [1]. The FDA-approved version is authorized only for use in Clinical Laboratory Improvement Amendments (CLIAs) [93]. In parallel, amplification-free CRISPR-based assays, although previously discussed, remain limited by their relatively low sensitivity compared to amplification-based methods. This remains a critical bottleneck for clinical translation. Consequently, the further engineering of Cas enzymes, improvement of crRNA-target recognition efficiency, and integration with highly sensitive readout systems are essential to enhance detection performance without compromising simplicity or portability. Lyophilization has emerged as a promising strategy for supporting the commercialization and field deployment of CRISPR-Cas-based diagnostics, particularly in resource-limited settings where the cold chain presents logistical and economic barriers [94,95]. In solutions, Cas enzymes and guide RNAs are highly sensitive to degradation, including protein denaturation by precipitation, aggregation, oxidation, and deamidation [94,95], and RNA degradation via RNases or hydrolysis [96]. Although ultra-low temperature storage can slow degradation, it does not prevent damage caused during freezing, such as ice-induced concentration effects that lead to the aggregation and loss of functionality, a phenomenon known as “freeze-concentration” [94,95]. Lyophilization addresses these challenges by stabilizing labile reagents through dehydration, thereby preserving structural and functional integrity during transport and storage [97]. The process comprises three key stages: rapid freezing below the triple point, primary drying via sublimation under reduced pressure, and secondary drying (desorption) to remove residual bound water [97]. Nevertheless, this technique exposes biological components to intense stress, potentially resulting in irreversible inactivation [9]. To counteract this, various excipients have been successfully employed [9]. Non-reducing disaccharides, such as trehalose and sucrose, stabilize proteins by forming hydrogen bonds and preserving the hydration shell, reducing damage during desorption [9]. For RNA, lyophilization in nuclease-free water alone significantly reduces recovery rates, whereas formulations with 10% trehalose maintain high recovery after storage at 4 °C for 10 months [98]. Bulking agents like mannitol or glycine help maintain structural matrix integrity during drying [98], while non-ionic surfactants such as Tween 20 mitigate aggregation during rehydration [9]. Optimizing lyophilization requires the careful control of formulation parameters. Increasing protein concentrations, reducing freezing rates to promote larger ice crystal formation, and using buffering agents like Tris that resist pH shifts during freezing have all been shown to mitigate protein denaturation at the ice–water interface [98,99]. Although most CRISPR-based diagnostic systems remain in solution form, a limited number of studies have demonstrated the feasibility of lyophilized formats. Several studies have investigated the impact of lyophilization on CRISPR-based diagnostics, highlighting both challenges and promising strategies for maintaining reagent stability. In the SHINEv2 platform, enzyme activity was significantly reduced after lyophilization but partially restored through the addition of stabilizing agents such as sucrose and mannitol and by removing destabilizers like PEG and KCl [9]. This combined approach retained most of the original activity; however, diagnostic performance remained suboptimal at low target concentrations, and room-temperature storage led to rapid activity loss, likely due to the absence of surfactants that prevent protein aggregation. In contrast, the SHERLOCK platform reported enhanced sensitivity post-lyophilization without the addition of stabilizers, an effect attributed to increased sample input volumes during resuspension [100]. A further evaluation of a lyophilized CRISPR-Cas12 assay for SARS-CoV-2 detection demonstrated that lyophilized reagents pre-loaded as beads in eight-strip tubes enabled simplified handling, requiring only the addition of PCR-grade water and RNA extract [101]. The assay maintained high diagnostic accuracy with near-perfect concordance to RT-qPCR, while offering room-temperature stability (15–30 °C), cost-effectiveness, and suitability for low-resource settings [101]. In a more recent study, test reagents were optimized using saccharide-based protectants, including trehalose, pullulan, and mannitol, to enhance reagent stability. The results revealed that while all three agents conferred protective effects, pullulan carried out the superior preservation of enzymatic activity. For RPA-based detection, a formulation of 2% trehalose, 5% pullulan, and 20% mannitol was found optimal. Meanwhile, for CRISPR-Cas12 assays, the most effective composition included 10% trehalose, 5% pullulan, and 10% mannitol, ensuring enhanced reagent stability and functionality under variable light and storage conditions [102]. Together, these findings underscore the potential of lyophilization when paired with tailored excipient strategies to facilitate the deployment of CRISPR-based diagnostics in decentralized and resource-limited environments.

The analysis of keyword co-occurrence is a valuable tool for identifying major themes and tracking research trends over time [103]. This method is essential for highlighting key topics in scientific work, defining specific study areas, and enhancing the visibility of research articles. Keywords, as a navigational tool, facilitate the identification and integration of relevant works while offering a better understanding of research priorities in a given field. In our bibliometric analysis, Figure 8 illustrates the most frequently repeated keywords (at least five occurrences) in scientific publications, revealing the primary areas of interest surrounding CRISPR-Cas systems applied to viral diagnostics. The dominant cluster, centered on the “CRISPR-Cas system”, reflects its pivotal role in current research, underscoring the growing importance of this technology in detecting viral infections. Associated terms such as “virus detection”, “molecular diagnosis”, and “point-of-care testing” highlight the focus on rapid and accessible applications, particularly for resource-limited settings. The red clusters, encompassing keywords like “SARS-CoV-2” and “COVID-19”, emphasize the surge in research between 2019 and 2022, a period marked by the pandemic crisis. These keywords reflect the substantial global efforts to optimize diagnostic tools, as evidenced by terms such as “nucleic acid amplification” and “molecular diagnosis”, which demonstrate the commitment to developing effective diagnostic solutions for SARS-CoV-2. Furthermore, emerging innovations are evident in specific clusters related to “electrochemical detection”, “biosensors”, “metal nanoparticles”, and “smartphones”, showcasing the technological convergence aimed at enhancing diagnostic sensitivity and portability. These advancements, particularly in settings without complex infrastructures, highlight the growing interest in integrating modern and accessible solutions into CRISPR-Cas systems. Finally, a particularly relevant keyword, “monkeypox virus”, underscores the scientific community’s responsiveness to emerging health crises. The rapid detection of monkeypox using CRISPR-Cas systems illustrates the adaptability of these technologies to diverse contexts and pathogens. This flexibility demonstrates the multidimensional potential of CRISPR-Cas systems to address current diagnostic challenges while reinforcing their utility in an expanding range of applications.

Beyond all these advantages and despite the remarkable potential of CRISPR-Cas technologies, a critical aspect that must be addressed is the comparison between standard diagnostic methods such as quantitative PCR (qPCR) and emerging CRISPR-Cas-based detection platforms. According to the most cited article identified in our bibliometric analysis [47], the reported limit of detection (LoD) for a CRISPR-Cas-based assay was 10 copies/μL of an input sample. In contrast, the CDC’s SARS-CoV-2 RT-qPCR assay achieved lower detection limits, with values as low as 1 copy/μL (input b) and 3.2 copies/μL (input c), along with a clinical sensitivity of 95% and specificity of 100%. These discrepancies can be attributed, in part, to the fact that early studies involving CRISPR-based diagnostics in 2020 were still in the optimization phase, as various research teams were working to improve performance through engineering of Cas proteins, crRNA design, and signal amplification strategies. Recent comparative studies have further clarified the evolving landscape of nucleic acid detection technologies. One such study assessed the detection limits of several methods, including conventional PCR, quantitative PCR (qPCR), loop-mediated isothermal amplification (LAMP), and RPA coupled with CRISPR/Cas12a. The results demonstrated that conventional PCR achieved a detection threshold of 1.00 × 10^0^ ng/μL and qPCR reached 1.00 × 10^−1^ ng/μL, while both LAMP and RPA-Cas12a displayed improved sensitivity, detecting nucleic acids down to 1.00 × 10^−2^ ng/μL [40]. Despite these promising developments, it is important to note that surpassing the sensitivity of qPCR remains technically feasible, but it often comes at a higher cost. Achieving superior performance typically requires the integration of advanced reagent chemistries, sophisticated signal amplification systems, or more complex instrumentation. As a result, there exists an inherent trade-off: While low-cost CRISPR-based assays may compromise sensitivity, efforts to increase analytical performance inevitably raise production costs. This balance between affordability and performance highlights the need for continuous innovation in molecular design and platform engineering to ensure that CRISPR-based diagnostics can eventually rival or exceed qPCR not only in sensitivity and specificity but also in scalability, accessibility, and practical deployment. Furthermore, it is crucial to consider the nature of assay outputs. Most CRISPR-based detection systems currently provide qualitative results indicating the presence or absence of a target nucleic acid, whereas qPCR platforms, such as the CDC’s SARS-CoV-2 qRT-PCR, deliver quantitative outputs that inform viral load. Transforming CRISPR-based assays into quantitative formats would require the integration of additional calibration curves, precise reaction control, and potentially real-time fluorescence monitoring, all of which would significantly increase cost and technical complexity. While such a setup may be feasible for use at point-of-care facilities, it could hinder the development of low-cost, home-based, or over-the-counter CRISPR diagnostics due to constraints in portability and affordability

## 5. Conclusions

The remarkable progress in CRISPR-Cas research between 2019 and 2024 highlights its immense potential to revolutionize the diagnosis of viral infections. With numerous advantages over traditional methods, this innovative technology stands out as a promising solution capable of transforming point-of-care testing in the near future. The integration of artificial intelligence; the development of new chemical approaches; and advancements in automation, lyophilization, and the engineering of fluidic platforms for multiplexing pave the way for major innovations in portable self-testing and the implementation of POCT (point-of-care testing) devices. These combined efforts could establish CRISPR-Cas as an essential alternative to accelerate and simplify viral diagnostics.

## 6. Strengths and Limitations

To the best of our knowledge, this is the first literature review and bibliometric analysis focused on global research trends concerning the use of the CRISPR-Cas system as a diagnostic method for viral infections. Although a previous bibliometric analysis explored the use of CRISPR-Cas12 and Cas13 in the detection of infectious diseases in general, our study specifically focuses on viral infections while also including other Cas proteins, such as Cas9, Cas14, and dCas (DeadCas). Furthermore, Samson Léta et al. [41] included only articles published between 2015 and 2022, whereas our study extends the analysis from 2019 to 2024. This extension provides a continuation of the work and highlights the ongoing impact of CRISPR-Cas systems in the diagnosis of viral infections, particularly in 2024, when the outbreak of monkeypox gained the attention of the World Health Organization (WHO). We also explored the most recent publications in this field and highlighted a limitation commonly noted in previous bibliometric studies: the reliance on a single database, typically Web of Science (WOS); this may result in the omission of significant studies [1]. To address this issue, we conducted our bibliometric analysis using three different databases.

However, our study has certain limitations. We only included open access articles, and paywalled publications were excluded due to the lack of institutional access. This exclusion might have led to the omission of relevant studies. Additionally, the focus on viral infections, while providing specificity to the study, may limit the overall scope of the analysis.

## Figures and Tables

**Table 1:** Top 10 most cited articles in the field of CRISPR-based viral infection detection.

Paper	DOI
Broughton JP, 2020, NAT BIOTECHNOL [47]	10.1038/s41587-020-0513-4
Kellner MJ, 2019, NAT PROTOC [48]	10.1038/s41596-019-0210-2
Fozouni P, 2021, CELL [49]	10.1016/j.cell.2020.12.001
Ding X, 2020, NAT COMMUN [50]	10.1038/s41467-020-18575-6
Ackerman CM, 2020, NATURE [51]	10.1038/s41586-020-2279-8
Patchsung M, 2020, NAT BIOMED ENG [52]	10.1038/s41551-020-00603-x
Li L, 2019, ACS SYNTH BIOL [53]	10.1021/acssynbio.9b00209
Arizti-sanz J, 2020, NAT COMMUN [54]	10.1038/s41467-020-19097-x
Freije CA, 2019, MOL CELL [55]	10.1016/j.molcel.2019.09.013
Huang Z, 2020, BIOSENS BIOELECTRON [56]	10.1016/j.bios.2020.112316

**Table 2:** Analysis of the top 10 articles on the use of CRISPR for viral infection detection.

Paper	CRISPR Protein /Efector Protein	Method	Target Type/Target Virus	Amplification Method	Assay Time (Minutes)	Sample Type	Steps	Complementary Technology Used	Sensitivity (LoD)	Specificity	Stability and Portability
BROUGHTON JP, 2020, NAT BIOTECHNOL [47]	CRISPR-Cas12a	CRISPR-based DETECTR assay (DNA Endonuclease-Targeted CRISPR Trans Reporter)	RNA SARS-CoV-2	RT-LAMP (reverse-transcription loop-mediated isothermal amplification)	30–40	Nasopharyngeal and oropharyngeal swabs (respiratory swabs)	Two-step process (RT-LAMP for amplification, followed by Cas12 detection)	Lateral flow strips and fluorescence-based detection	10 copies per µL	High specificity 95% positive 100% negative	YES
KELLNER MJ, 2019, NAT PROTOC [48]	CRISPR-Cas13a	(Specific High-Sensitivity Enzymatic Reporter UnLOCKing) SHERLOCK	RNA Zika virus, Dengue virus	RT-RPA (reverse transcription-recombinase polymerase amplification)	Less than 60	Respiratory swabs (nasopharyngeal or oropharyngeal)	Two-step procedure (pre-amplification with RPA followed by CRISPR-Cas13 detection)	Lateral flow assay or fluorescence-based detection	~50 fM	High (can distinguish between single-nucleotide variants)	YES
FOZOUNI P, 2021, CELL [49]	CRISPR-Cas13a		RNA SARS-CoV-2	No amplification required (amplification-free detection)	30	Nasal swabs	Single-step process	Mobile phone-based fluorescence microscope for signal readout	As low as 100 copies/mL	High (tested against other respiratory viruses like HCoV-NL63, HCoV-OC43, and MERS-CoV, with no cross-reactivity detected)	YES
DING X, 2020, NAT COMMUN [50]	CRISPR-Cas12a	All-In-One Dual CRISPR-Cas12a (AIOD-CRISPR)	RNA SARS-CoV-2	RPA (recombinase polymerase amplification)	40	Respiratory swabs	One-pot reaction (single step for both amplification and detection)	Fluorescence and lateral flow detection (low-cost hand warmer used as an incubator for point-of-care testing)	Down to ~5 copies	High	YES
ACKERMAN CM, 2020, NATURE [51]	CRISPR-Cas13a	Combinatorial Arrayed Reactions for Multiplexed Evaluation of Nucleic Acids (CARMEN)	DNA/RNA SARS-CoV-2, Influenza A strains, HIV drug-resistance mutation, other 169 human-associated viruses	PCR (polymerase chain reaction) and RT-PCR and recombinase polymerase amplification (RPA)	Several hours	Throat and nasal swab samples Plasma and serum from patients	Two-step process	Fluorescence microscopy for detection Color coding for identifying sample-droplet pairs	Attomolar sensitivity	High	YES
PATCHSUNG M, 2020, NAT BIOMED ENG [52]	CRISPR-Cas13a	(Specific High-Sensitivity Enzymatic Reporter UnLOCKing) SHERLOCK	RNA SARS-CoV-2	RT-RPA (reverse transcription-recombinase polymerase amplification)	<120	Nasopharyngeal and oropharyngeal swabs	Multi-step process	Fluorescence-based detection, with an option for lateral flow readout	42 copies per µL	High 100%	YES
LI L, 2019, ACS SYNTH BIOL [53]	CRISPR-Cas12b	One-hour Low-cost Multipurpose Highly Efficient System (HOLMESv2)	DNA/RNA Japanese encephalitis virus (JEV)	LAMP (Loop-mediated isothermal amplification), RT-LAMP (reverse-transcription loop-mediated isothermal amplification)	<60	DNA, RNA (including body fluids like urine)	One-step process (integration of amplification and detection)	Fluorescence detection and lateral flow assay	As low as 10^−8^ nM for DNA and RNA	High (distinguishes single-nucleotide polymorphisms (SNPs))	YES
ARIZTI-SANZ J, 2020, NAT COMMUN [54]	CRISPR-Cas13a	Streamlined Highlighting of Infections to Navigate Epidemics (SHINE)	RNA SARS-CoV-2	RPA (recombinase polymerase amplification)	50	Nasopharyngeal swabs and saliva	Single-step process	Fluorescent readout and companion smartphone application for result interpretation	100 copies per µL	High 100%	YES
FREIJE CA, 2019, MOL CELL [55]	CRISPR-Cas13a, CRISPR-Cas13b	Cas13-assisted Restriction of Viral Expression and Readout (CARVER)	Single-stranded RNA (ssRNA) viruses, including lymphocytic choriomeningitis virus (LCMV), influenza A virus (IAV), and vesicular stomatitis virus (VSV)	RT-RPA (reverse transcription-recombinase polymerase amplification)	120	Viral RNA extracted from cell culture supernatants	Multi-step process	SHERLOCK platform for detecting viral RNA after Cas13-mediated cleavage	10 copies per µL	High 100%	
HUANG Z, 2020, BIOSENS BIOELECTRON [56]	CRISPR-Cas12a	COVID-19 CRISPR-FDS	RNA SARS-CoV-2	RT-RPA (reverse transcription-recombinase polymerase amplification)	50	Nasal swabs	One-step process (for RNA extraction, target amplification, and fluorescent detection)	Fluorescent signal detection using SpectraMax i3x Multi-Mode Microplate Reader	As low as 2 copies	Low 71.4% (it showed some additional detections that the qPCR missed)	YES

**Table 3:** Comparison of distinct features of Cas12 and Cas13 nuclease.

Feature	Cas12	Cas13
Target Molecule	Double-stranded DNA (dsDNA)	Single-stranded RNA (ssRNA)
Commercial Availability	Yes	Yes
tracrRNA Required	No	No
crRNA Structure	Single crRNA (20 nt spacer + 21 nt repeat)	Single crRNA (28–30 nt spacer + direct repeat)
crRNA Length (Mature)	42–44 nucleotides	64–66 nucleotides (depends on subtype)
PAM or PFS Requirement	Requires PAM (TTTV)	No PAM; requires PFS (H, any base except G at 3′ end)
Complementarity	crRNA complementary to opposite strand containing PAM	crRNA complementary to target RNA
Endonuclease Domain	RuvC-like domain	HEPN domains
Cleavage Type	Double-stranded DNA cleavage	Single-stranded RNA cleavage
Cleavage Site	~18 nt from 3′ PAM strand + ~23 nt from 5′ opposite strand	Specific site (PFS) + collateral cleavage of non-target RNAs
Seed Region	Well characterized (first 6–8 nt), tolerant to SNPs	Not clearly defined; mismatch sensitivity varies among subtypes
Collateral Activity	Yes	Yes
Trans Cleavage Activity	Yes, but limited	Yes
Multiplexing Capability	Yes, easy and functional	Yes, easy and functional
Sensitivity	High	High
Cost	Low cost (usually)	Low cost (usually)
Origin	Prokaryotes	Prokaryotes
Methylated DNA Binding	Not detected	Not studied

## Data Availability

All data generated or analyzed during this study are included in this published article.
